# Tracheotomy in Apoplexy and Epilepsy

**Published:** 1853-06

**Authors:** 


*Tracheotomy in Apoplexy and Epilepsy.*—Dr. Marshall Hall has lately proposed the operation of tracheotomy as a therapeutic measure in certain cases of apoplexy and epilepsy. It deserves notice, to say the least, as one of the passing curiosities of the day. More than this cannot be said of it, till facts are brought forward to afford some evidence of its value. It may be doubted whether the speculative grounds for confidence in its propriety will be deemed sufficient, with many minds, to justify the acquisition of experimental facts. By an article contained in the Eclectic Department of our last No., it will be perceived that Dr. Green, of New York, proposes to substitute cauterization of the larynx for tracheotomy. This measure is devoid of the moral objections to that suggested by Marshall Hall, but with what success it may be practiced remains to be determined by repeated observations. A consideration to be borne in mind in resorting to these, or any other active measures in epilepsy is this—a variety of perturbatory influences are found to exert a temporary effect in suspending the paroxysms, without proving in any degree permanently curative. In this way are to be explained a portion of the reputed cures of this disease effected by numerous medicinal agents. An unusually long interval between the paroxysms occurs, and, in the mean time, either the patient passes from observation, or the practitioner hastens to announce his success, and does not correct the mistake when he subsequently discovers it.

In the absence of sufficient evidence either for, or against the singular mode of treatment recommended by Marshall Hall, we have not the presumption to offer any opinion respecting its merits. For the information of our readers we quote the following letter communicated for the London Lancet:

*Laryngismus* is the event which separates the *graver* from the *milder* forms of apoplexy and of epilepsy, assuming in the former the *paralytic*, in the latter the *spasmodic* form.

It is to avert the effects of this laryngismus that I propose to institute tracheotomy. This is my object, and has been so from the beginning. I have never had the idea of proposing empirically a remedy for the *name* of a disease, be it apoplexy or epilepsy. In order that my view may be carried out fairly, the following facts must be first established:

1. The case must still be inorganic; there must be a well founded *hope* of doing good.

2. There must be the *dignus vindice nodus;* there must be sufficient reason for adopting the measure; there must be *danger* to life, mind, or limb.

3. This danger must depend on *laryngismus;* that is, on paralytic laryngeal dyspnœa or stertor, in the apoplectic or comatose cases, and on spasmodic laryngeal dyspnœa in the epileptic.

In regard to all these circumstances, a just and sufficient *diagnosis* must be established, as in all other cases in the practice of medicine. Without such diagnosis the remedy would be, must be, employed in vain, as it would assuredly be instituted without sufficient reason.

Every event convinces me that, if these just precautions be effectually adopted, the success of the remedy will prove infallible; and that the cases are numerous in which life and intellect will be saved.

*If the remedy prove ineffectual, it will be because the* DIAGNOSIS *has been inadequate.*

I am, sir, your obedient servant,

MARSHALL HALL.

